# Effect of Case-Based Learning combined with Flipped Classroom on residents’ axillary vein puncture and catheterization ability

**DOI:** 10.3389/fmed.2026.1747024

**Published:** 2026-03-25

**Authors:** Pengchao Guo, Yanan Zhao, Lan Ying, Shouyin Jiang, Li Wang

**Affiliations:** 1 Department of Emergency Medicine, The Second Affiliated Hospital of Zhejiang University School of Medicine, Hangzhou, China; 2 Department of Ultrasound Medicine, The Second Affiliated Hospital of Zhejiang University School of Medicine, Hangzhou, China

**Keywords:** axillary vein puncture and catheterization, Case-Based Learning, Entrustable Professional Activities, Flipped Classroom, Mini-CEX

## Abstract

**Objective:**

To evaluate the efficacy of the Case-Based Learning (CBL) combined with Flipped Classroom (FC) teaching model in training resident physicians to master axillary vein puncture and catheterization (AVPC) skills, and to provide an optimized teaching strategy for clinical skill training in critical care medicine.

**Methods:**

This was a randomized controlled study conducted on 93 resident physicians rotating in the intensive care unit from September 2023 to September 2024. Participants were randomly assigned to the reform group (*n* = 46) and the control group (*n* = 47) via a random number table method. The reform group adopted the CBL-FC integrated teaching model, while the control group received conventional traditional teaching. After training, the Entrustable Professional Activities (EPA) scale was used to assess AVPC competence, and the two groups were compared in terms of AVPC first puncture success rate, operation time, Mini-Clinical Evaluation Exercise (Mini-CEX) scores, and EPA compliance rate. A questionnaire survey was also conducted to evaluate residents’ satisfaction with the teaching mode.

**Results:**

The reform group had a significantly higher first puncture success rate and shorter operation time than the control group (*t* = 6.474, 6.459, all *p* < 0.05). Mini-CEX scores in the domains of operational skills, clinical judgment, and self-reflection & learning of professional attitude were markedly higher in the reform group (all *p* < 0.05). The EPA compliance rate of the reform group was significantly superior to that of the control group (*χ*^2^ = 24.373, *p* < 0.05). Additionally, the reform group showed significantly higher satisfaction with course content, simulation practice, classroom interaction, teaching methods and course resources (all *p* < 0.05).

**Conclusion:**

The CBL-FC integrated teaching model exerts a remarkable educational effect on AVPC training for residents, effectively improving their clinical operational proficiency, clinical decision-making ability and learning engagement. It serves as a feasible and effective optimized teaching approach to replace traditional teaching, and can be popularized in clinical skill training for residents in critical care and emergency medicine, which provides a valuable reference for the reform of clinical teaching models in related medical fields.

## 1 Introduction

Axillary vein puncture and catheterization (AVPC), a core central venous catheterization technique, is the preferred approach for trauma patients and requires precise, standardized operation plus effective complication prevention (1). Resident physicians in critical care must master both solid foundational knowledge and robust clinical judgment and operational skills for AVPC, yet traditional AVPC training—combining theoretical lectures with clinical practice—suffers from unquantifiable outcomes, low learner engagement and limited practical time, hindering the improvement of hands-on skills (2). Innovative medical education models have gained increasing attention, with Case-Based Learning (CBL) and Flipped Classroom (FC) proving effective for enhancing clinical reasoning and practical skills. CBL cultivates clinical decision-making via group discussion of real cases (3), while FC shifts knowledge acquisition to pre-class self-study, allocating in-class time for discussion, practice and interaction to boost participation (4). But their integrated application in the training of invasive procedural skills like AVPC in critical care medicine remains largely underexplored. Most relevant studies either focus on the single application of CBL or FC, or target non-invasive clinical skills training, with scarce empirical evidence on the efficacy of their combination for AVPC training. Additionally, existing AVPC training research lacks a comprehensive, competency-oriented assessment framework; while Entrustable Professional Activities (EPAs) and Mini-Clinical Evaluation Exercise (Mini-CEX) have become gold-standard tools for objective assessment of residents’ clinical competencies (5–7), their combined use to evaluate the effect of innovative teaching models on AVPC training is rarely reported. We hypothesize that: (1) the CBL-FC model will significantly increase the first puncture success rate and shorten the operation time of residents in AVPC practice; (2) residents receiving the CBL-FC model will achieve higher scores in Mini-CEX (especially in operational skills and clinical judgment domains) and a higher EPA compliance rate for independent AVPC performance; (3) the CBL-FC model will significantly improve residents’ satisfaction with AVPC training by enhancing active learning engagement. Therefore, this study aims to explore the application efficacy of the CBL-FC integrated teaching model in AVPC resident training using EPA and Mini-CEX as the core assessment tools, and to provide an evidence-based optimized teaching strategy for invasive procedural skill training in critical care and emergency medicine.

## 2 Materials and methods

### 2.1 General information

This study selected 93 resident physicians rotating in the intensive care unit of our department from September 2023 to September 2024. We used the random number table method for group assignment: two research assistants numbered the 93 eligible residents 1 to 93 by their rotation enrollment order, generated a random number table with SPSS 23.0, and assigned them to the reform and control groups at a 1:1 ratio (odd numbers for the reform group, even numbers for the control group). For allocation confidentiality, grouping results were stored in password-protected electronic files and sealed paper envelopes marked only with resident serial numbers, which were opened only at the start of formal teaching intervention. All researchers involved in teaching and assessment were kept blind to the grouping results throughout the study. The CBL/FC teaching model was used in the reform group, and the traditional teaching model was used in the control group. The reform group comprised 46 participants, including 27 males, with an average age of (24.52 ± 3.54) years; the control group had 47 participants, including 25 males, with an average age of (24.43 ± 3.36) years (Table 1). There were no statistical differences in general information between the two groups, indicating comparability.

**Table 1:** General information results.

Clinical parameter		Reform group (*n* = 46)	Control group (*n* = 47)	*χ*^2^ value	*p* value
Gender	Male/female	27/19	25/22	0.286	0.593
Grade	First grade	15 (32.61)	24 (51.06)	3.599	0.165
	Second grade	20 (43.48)	13 (27.66)		
	Third grade	11 (23.91)	10 (21.28)		
Educational background	Graduate	12 (26.09)	13 (27.66)	0.029	0.864
	Undergraduate	34 (73.91)	34 (72.34)		
Profession	Emergency	21 (45.65)	18 (38.30)	0.561	0.472
	Non-emergency	25 (54.35)	29 (61.70)		

Inclusion criteria: (1) full participation in the AVPC training course; (2) complete attendance in teaching activities and assessments during the rotation; (3) first rotation in the emergency department; (4) no training in AVPC within the last year.

Exclusion criteria: (1) suspension of emergency department rotation due to personal leave, sick leave, maternity leave, etc.; (2) incomplete participation in emergency specialty teaching activities and basic skills training; (3) resident physicians with depression or other mental illnesses that prevent them from completing clinical practice operations; (4) non-participation in the questionnaire survey.

### 2.2 Research methods

#### 2.2.1 The teaching process of reform group

Pre-class preparation: The instructor will distribute course materials, clarify the learning objectives and teaching content, covering axillary venous anatomy, puncture techniques and steps, prevention of complications, and clinical case data. Residents are required to conduct self-study in advance, including reading relevant literature, watching puncture operation videos, and participating in online tests to assess their learning progress. Additionally, the instructor will assign specific learning tasks, requiring residents to complete pre-class reading, self-testing, and related self-assessment questionnaires to help them understand the theoretical framework and operational standards for AVPC.

In-class instruction: The instructor will begin by introducing specific clinical cases to stimulate discussion among the residents, focusing on analyzing puncture site selection, preparation, operational challenges, and methods for managing complications. Residents will be divided into groups to discuss and deepen their clinical decision-making through questioning. Subsequently, the instructor will provide a detailed knowledge explanation based on the case and demonstrate the standard steps and key techniques of AVPC using a model. Next, residents will practice simulated operations in groups, with the instructor providing real-time feedback and correcting errors during the procedure. Peer interaction will further enhance residents’ operational skills. Finally, the instructor will offer immediate evaluations during the observation of the operation and encourage residents to ask questions and engage in group discussions to deepen their understanding of AVPC.

Clinical instruction: The instructor must ensure that the residents have mastered the basic skills of AVPC. Under the instructor’s guidance, residents will carry out clinical practice, with the instructor observing in real time and providing operational guidance. After the procedure, the instructor will provide feedback based on the residents’ performance, assess strengths and weaknesses, and offer personalized tutoring to improve their skills.

#### 2.2.2 The teaching process of traditional group

Pre-class preparation: The instructor will determine the teaching theme based on the course syllabus and actively prepare the lesson. A specialized lecture will be given according to the teaching requirements, covering key knowledge points and learning objectives consistent with the reform group.

In-class instruction: The class will primarily include theoretical lectures and simulated operations. The theoretical knowledge will cover basic knowledge, anatomical structures, indications and contraindications, relevant tools and materials, and specific steps for AVPC. Residents will attend the lecture attentively and raise any questions, which the instructor will answer diligently. The instructor will also conduct hands-on practice using models, allowing residents to simulate the puncture process and practice the puncture techniques and operational maneuvers.

Clinical instruction: The clinical teaching process will be the same as that in the reform group.

### 2.3 Observation indicators

#### 2.3.1 Success rate of puncture and operation time

The success rate of puncture refers to the proportion of residents in each group who successfully complete the axillary venous puncture in a real-world setting, relative to the total number of residents in the group. The operation time refers to the time taken by the residents from the start of disinfection to the completion of catheter fixation after the puncture.

#### 2.3.2 Mini-CEX evaluation

Residents were assessed by three independent attending physicians who were blinded to the group allocation and not involved in the teaching intervention of this study during AVPC procedures on real patients. The Mini-CEX utilized a three-tier, nine-point scale: needs improvement (1–3 points), satisfactory (4–6 points), and excellent (7–9 points), evaluating knowledge mastery, procedural skills, clinical judgment, and professional attitude across 11 items (7). This Mini-CEX scale has been validated for good reliability and validity. Consistent with the excellent internal consistency reliability demonstrated in this study (Cronbach’s *α* = 0.92–0.97), previous studies reported a Cronbach’s *α* coefficient of 0.89 for internal consistency, along with a content validity index (CVI) of 0.92 (8).

#### 2.3.3 EPAs compliance rate

The ability of AVPC in the real scenario was evaluated by the teachers through EPAs (Table 2). EPAs compliance rate was defined as the proportion of residents with EPAs ≥ 4 points. The EPA assessment scale for invasive vascular procedures has been established and validated in the Chinese resident training system, with good construct validity (confirmatory factor analysis: *χ*^2^/df = 2.13, RMSEA = 0.07) and inter-rater reliability (ICC = 0.85) in local emergency medicine training assessment (5, 6). In our hospital, this scale has been applied in clinical skill assessment for emergency residents for 3 years, with a verified internal consistency Cronbach’s *α* of 0.87.

**Table 2:** Entrustable Professional Activities level.

Level	Content
1	Residents cannot perform AVPC under the direct supervision of their superior physicians.
2	Residents perform AVPC under the full, direct and active supervision of superior physicians.
3	Residents perform AVPC under the passive supervision of superior physicians.
4	Residents can perform AVPC independently.
5	Residents can supervise and instruct others to perform AVPC.

#### 2.3.4 Resident satisfaction survey

Feedback was collected from residents via a questionnaire to assess the overall effectiveness of the teaching process. This 8-item questionnaire covered four dimensions including teaching content, teaching method, teaching effect and learning satisfaction, and was developed based on relevant literature research and the characteristics of CBL-FC integrated teaching. A pilot test was conducted on 30 resident physicians not included in the formal study, with all item-total correlation coefficients ≥0.40 and no items deleted after the test. The questionnaire was verified to have good internal consistency reliability (Cronbach’s *α* = 0.87) and content validity (CVI = 0.90). Anonymous questionnaire collection and a standardized 5-point Likert scale (1 = very dissatisfied, 5 = very satisfied) were adopted to ensure the objectivity of the survey results (9).

### 2.4 Statistical methods

In this study, it was assumed that there would be a significant difference between the two groups in terms of improvement in axillary venous puncture skills, and conventional statistical methods were used for sample size estimation. Considering that a two-tailed test was used, the significance level (*α*) was set to 0.05, and the test power was set to 80%. Based on the expected effect size, the calculation indicated that at least 45 participants per group would be required. SPSS 23.0 was used to calculate the reliability of the scale. Continuous data that followed a normal distribution were expressed as mean ± standard deviation. Count data were described as (*n*, %), and intergroup differences in continuous data were compared using t-tests, while chi-square tests were used for count data comparisons. Intraclass correlation coefficient (ICC) was used to test the inter-rater reliability of Mini-CEX and EPA scores among the three assessors, with ICC > 0.8 considered as good inter-rater reliability. A *p*-value < 0.05 was considered statistically significant.

## 3 Results

### 3.1 Comparison of puncture success rates and operation times

The success rate of puncture in the reform group was also significantly higher than that in the control group, and the operation time was significantly lower than that in the control group (*p* < 0.05), as shown in Table 3.

**Table 3:** Comparison of puncture success rate and operation time.

Outcome indicators	Reform group (*n* = 46)	Control group (*n* = 47)	*t* value	*p* value
First puncture success rate (*n*, %)	36 (78.26)	25 (53.19)	6.474	0.011
Operating time (min)	3.61 ± 0.79	7.08 ± 3.59	6.459	0.001

### 3.2 Comparison results of the Mini-CEX scores and EPAs

The scores of Mini-CEX in operation skills, clinical judgment and professional attitude in the reform group were significantly higher than those in the control group (*p* < 0.05). The EPAs compliance rate of the reform group was significantly higher than that of the control group (*p* < 0.05), as shown in Table 4.

**Table 4:** Comparison results of Mini-CEX scores and EPAs compliance rates.

Mini-CEX evaluation dimension		Reform group (*n* = 46)	Control group (*n* = 47)	*t* value	*p* value
Knowledge mastery	Understanding of theoretical knowledge	6.17 ± 1.77	6.06 ± 1.80	0.298	0.767
	Anatomical knowledge of the axillary vein	6.70 ± 0.47	6.64 ± 0.49	0.581	0.562
Operational skill	Preparation of materials	8.50 ± 0.59	7.49 ± 0.62	8.062	<0.001
	Personal preparation	8.28 ± 0.58	7.57 ± 0.68	5.368	<0.001
	Patient preparation	7.46 ± 0.59	6.89 ± 0.73	4.100	<0.001
	Puncture technique (needle Angle, depth)	7.80 ± 0.54	6.68 ± 0.69	8.679	<0.001
	Techniques for Venipuncture/Catheterization	6.74 ± 0.44	6.13 ± 0.34	7.489	<0.001
Clinical judgment	Identification and management of complications	7.13 ± 0.75	5.96 ± 0.83	7.138	<0.001
	Assessment of indications and contraindications	6.91 ± 0.81	6.06 ± 0.60	5.730	<0.001
Professional attitude	Awareness of professional ethics	7.89 ± 0.97	7.64 ± 0.49	1.594	0.114
	Self-reflection and learning	8.50 ± 0.69	8.19 ± 0.68	2.170	0.033
Mini-CEX score		82.09 ± 2.98	75.32 ± 3.28	10.400	<0.001
EPAs compliance rate (*n*, %)		38 (82.61)	15 (31.91)	24.373	<0.001

### 3.3 Comparison of teaching satisfaction

The reform group reported significantly higher satisfaction levels regarding course content, simulation practice, classroom interaction and discussion, teaching methods, and course resources compared to the control group (*p* < 0.05), as indicated in Table 5.

**Table 5:** Comparison of teaching satisfaction.

Satisfaction dimensions	Reform group (*n* = 46)	Control group (*n* = 47)	*t* value	*p* value
Teaching content	4.23 ± 0.64	3.94 ± 0.60	2.349	0.021
Simulated operations	4.28 ± 0.58	3.57 ± 0.68	5.368	0.001
Classroom interaction and discussion	4.50 ± 0.59	3.49 ± 0.62	8.062	0.001
Teaching methods	4.46 ± 0.59	3.89 ± 0.73	4.100	0.001
Curriculum resources	4.20 ± 0.71	3.79 ± 0.69	3.257	0.002

## 4 Discussion

Currently, central venous catheterization for trauma patients is often limited by patient positioning, which complicates the routine performance of internal jugular and certain subclavian vein cannulations. In contrast, ultrasound-guided AVPC provides advantages such as accurate localization, rapid hemostasis, and ease of operation, making it a safe and effective alternative to traditional venous access methods (10). However, teaching related to AVPC primarily relies on traditional methods, which can lead to inadequate proficiency among resident physicians. This lack of proficiency results in insufficient mastery of essential techniques, such as confirming guidewire placement and vascular entry, contributing to a high first-puncture failure rate. EPAs are currently used as supplementary assessments in clinical competency evaluations during resident training (11), particularly in emergency medicine, which involves a diverse range of EPAs and related conditions (12, 13). Therefore, this study aims to enhance puncture success rates by optimizing teaching methods and incorporating the EPA scale to evaluate the operational capabilities of resident physicians.

This study compares and analyzes two training methods, highlighting significant limitations in the traditional approach. Firstly, many training programs lack targeted practical training, which results in limited opportunities for residents to practice in simulated environments. This insufficient practice can lead to a lack of confidence and skill during actual procedures. Secondly, traditional training often emphasizes theoretical knowledge while neglecting the development of teamwork and emergency response skills, both of which are critical in clinical practice. Furthermore, the course content has not kept pace with advancements in medical technology, leading to a disconnect between training outcomes and actual clinical needs. Finally, residents typically experience low levels of engagement and interactivity during training, adversely affecting learning outcomes and overall satisfaction.

The combination of CBL and FC offers several advantages over traditional teaching methods. By integrating CBL and FC, residents gain a deeper understanding and practical application of puncture techniques in real clinical scenarios. This approach enhances their operational efficiency through interactive discussions and simulated exercises. Studies in fields like nephrology (14), ophthalmology (15), and pathology (16) have shown the effectiveness of the CBL/FC model, confirming its positive teaching outcomes. The CBL/FC model encourages active learning and independent exploration, where residents analyze real cases, engage in group discussions, and solve clinical problems, which leads to a better understanding of both theoretical knowledge and practical skills (3, 4)^.^ In contrast, traditional teaching methods often focus on one-way theoretical lectures, which lack sufficient interaction and problem-solving opportunities, resulting in slower improvements in clinical decision-making and skills. Additionally, the CBL/FC model emphasizes real-time feedback during simulations and group discussions, allowing supervisors to correct mistakes immediately based on residents’ performance (9). Traditional models, however, typically rely on teachers’ explanations and demonstrations, which do not provide the same level of personalized feedback and make it harder for residents to detect and correct errors in their operations.

Finally, the study results show that residents in the reform group were more satisfied with the course content and teaching methods compared to those in the control group, indicating that the CBL/FC training model better stimulates residents’ interest in learning and participation, thereby enhancing their overall training experience.

In this study, the Mini-CEX and EPA compliance rates were used to evaluate the teaching effect. These two evaluation methods have been widely used in clinical teaching assessments (17, 18). Mini-CEX emphasizes immediate feedback in real clinical environments, helping residents quickly identify and correct their shortcomings, and is particularly important in emergency teaching (17). On the other hand, a key feature of EPA is “progressive entrustment,” where residents gradually gain more independence and responsibility based on their ability during the learning process (17)^.^ Additionally, EPA has clear evaluation criteria, reducing subjective bias in the assessment from supervising teachers. Therefore, this comprehensive evaluation can provide a thorough and objective assessment of residents’ abilities in AVPC, while also offering more targeted and practical feedback to supervising teachers. Moreover, it provides a basis for improving the course content, thus enhancing the overall quality of teaching.

The limitations of this study are as follows: first, the sample size is relatively small and only covers residents in the intensive care unit, which makes it difficult to generalize the results to residents from other departments or hospitals and thus limits the applicability of the findings. Moreover, this study only assessed the training effects over a one-year period and failed to explore long-term outcomes or the sustained retention of skills. Additionally, the study adopted a single-blinded design: all assessors responsible for AVPC skill evaluation were strictly blinded to group allocation; however, residents were not blinded to the teaching model they received, which may have caused performance bias (e.g., higher learning motivation in the reform group). Furthermore, the satisfaction survey was completed by the residents themselves, and their unblinded status might have slightly affected the subjective satisfaction scores, though this bias was minimized through the use of a standardized 5-point Likert scale and anonymous questionnaire collection. In conclusion, the CBL/FC combined teaching model has significant advantages in AVPC, effectively improving residents’ clinical operation abilities and cognitive skills.

## Data Availability

The original contributions presented in the study are included in the article/supplementary material, further inquiries can be directed to the corresponding authors.
